# Title: Can changing the physical environment promote walking and cycling? A systematic review of what works and how

**DOI:** 10.1016/j.healthplace.2019.102161

**Published:** 2019-07

**Authors:** Jenna Panter, Cornelia Guell, David Humphreys, David Ogilvie

**Affiliations:** a MRC Epidemiology Unit, University of Cambridge, Cambridge, UK; b Centre for Diet & Activity Research (CEDAR), University of Cambridge, Cambridge, UK; c European Centre for Environment & Human Health, Medical School, University of Exeter, Exeter, UK; d Department of Social Policy and Intervention, University of Oxford, Oxford, UK; e Green Templeton College, University of Oxford, Oxford, UK

**Keywords:** Systematic review, Physical activity, Evaluation, Intervention, Environment, Urban design, Causality

## Abstract

Environmental changes aimed at encouraging walking or cycling may promote activity and improve health, but evidence suggests small or inconsistent effects in practice. Understanding how an intervention works might help explain the effects observed and provide guidance about generalisability. We therefore aimed to review the literature on the effects of this type of intervention and to understand how and why these may or may not be effective. We searched eight electronic databases for existing systematic reviews and mined these for evaluative studies of physical environmental changes and assessed changes in walking, cycling or physical activity. We then searched for related sources including quantitative or qualitative studies, policy documents or reports. We extracted information on the evidence for effects (‘estimation’), contexts and mechanisms (‘explanation’) and assessed credibility, and synthesised material narratively. We identified 13 evaluations of interventions specifically targeting walking and cycling and used 46 related sources. 70% (n = 9 evaluations) scored 3 or less on the credibility criteria for effectiveness. 6 reported significant positive effects, but higher quality evaluations were more likely to report positive effects. Only two studies provided rich evidence of mechanisms. We identified three common resources that interventions provide to promote walking and cycling: (i) improving accessibility and connectivity; (ii) improving traffic and personal safety; and (iii) improving the experience of walking and cycling. The most effective interventions appeared to target accessibility and safety in both supportive and unsupportive contexts. Although the evidence base was relatively limited, we were able to understand the role of context in the success of interventions. Researchers and policy makers should consider the context and mechanisms which might operate before evaluating and implementing interventions.

## Highlights

• Understanding how an intervention works might help explain the effects observed.
• We reviewed evidence for effects, contexts and mechanisms and assessed credibility.
• 6 out of 30 studies reported significant positive effects.
• Higher quality evaluations were more likely to report positive effects.
• Effective interventions targeted accessibility and safety in different contexts.

## 1 Introduction

Chronic diseases are the leading cause of death globally and many of these share common behavioural risk factors such as smoking, unhealthy diets, physical inactivity and excessive alcohol consumption. National and international organisations acknowledge that these behavioural risk factors have complex individual and social determinants (United Nations, 2018; World Health Organisation, 2013). Efforts to reduce the prevalence of some risk factors, such as smoking, have received substantial attention whilst physical inactivity has received less attention, even though the effects of inactivity on life expectancy and mortality are similar to those of smoking (Lee et al., 2012). Although physical activity has complex individual and social determinants, most approaches targeting individuals have had modest success (Reis et al., 2016).

A genuinely population-based public health strategy to promote physical activity would address the social determinants of activity, seeking to change the circumstances in which people live and the environments and policies that shape those circumstances such as employment, housing and transport (Panter and Ogilvie, 2016). These changes in the environment might target leverage points in the social and physical systems that generate and sustain patterns of inactivity such as the planning of towns and cities and the relative cost and convenience of different modes of transport (Panter and Ogilvie, 2016). These changes are endorsed by the Toronto Charter for Physical Activity which highlights urban design policies, transport systems and infrastructure as important areas for action (Global Advocacy Council for Physical Activity, 2010). Policymakers and practitioners are changing physical and social systems, but these changes often reflect civic common sense and are context-sensitive (Reis et al., 2016). Recent reviews of environmental interventions to promote physical activity identified small scale evaluative studies (Mayne et al., 2015; National Institute for Health and Care Excellence, 2018; Reis et al., 2016; Smith et al., 2017; Stappers et al., 2018), which comprised a mix of types of interventions (such as new greenspaces and transport infrastructure); evaluations varied widely in the approaches to dealing with confounding and how control groups were designed or implemented, and studied different population groups. Many of these studies also had questionable internal validity (Benton et al., 2016), bringing concerns about relevance and rigour to the fore. These and other reviews generally conclude that the evidence is “largely lacking” (Mayne et al., 2015), “inconclusive” (Stappers et al., 2018) and “indicative” (National Institute for Health and Care Excellence, 2018).

In 2015 Chris Whitty, the Chief Scientific Adviser for the UK Department of Health, wrote: “If the academic community could do one thing to improve the pathway from research to policy it would be to improve the status, quality, and availability of good synthesis” (Whitty, 2015). Good evidence synthesis is challenging and heterogeneity in primary studies is not unique to studies in this field (Becker et al., 2017) and may be perceived as a problem for those seeking to synthesise evidence. Typical reviews synthesise effects for broadly similar types or forms of interventions (National Institute for Health and Care Excellence, 2018) (e.g. cycle routes or greenspaces), but it is often difficult to provide evidence which supports more generalisable causal inference. Some authors have suggested that more nuanced ways of synthesising evidence are required to understand how and why interventions worked or did not work when applied in different contexts (Pawson, 2006; Petticrew, 2015). Others have suggested that complex interventions comprise key active components (Craig et al., 2008) and that the processes or mechanisms of an intervention act in the same way in different contexts and might be generalisable (Panter et al., 2017; Pawson, 2006). In other words, rather than synthesising evidence from similar classes or *forms* of interventions (e.g. cycle paths), it might be possible to synthesise evidence from interventions which have the same *function* (e.g. interventions which change the perceived safety of cycling regardless of the precise method used). This exploits the variation in contexts where similar (but not exactly the same) interventions have been implemented.

This approach fills two repeatedly raised evidence gaps: the first, about whether findings can be generalised to different settings (National Institute for Health and Care Excellence, 2018) and the second, about *how* interventions work, information that could strengthen the basis for causal inference (Victora et al., 2004). The second is particularly important for interventions that alter the environment, as these are unlikely to be allocated to places through a random process. A variety of study designs and methods (Vandenbroucke et al., 2016) will strengthen the case for causal inference, and such studies are likely to include assessments of both the strength of the causal estimation and the plausibility of the mechanisms (Vandenbroucke et al., 2016; Victora et al., 2004). Realist review logic draws on a range of types of evidence and a previous reviews of urban regeneration programmes suggest that safety and neighbourhood design mechanisms were important for walking using qualitative evidence (Kramer et al., 2017). However, as far as we are aware no reviews have demonstrated the applicability of this approach to environmental interventions to promote activity using quantitative and qualitative evaluative evidence.

We sought to use the case of environmental interventions which promote physical activity by directly targeting walking and cycling behaviours to embrace this heterogeneity and synthesise evidence in a way which supports more generalisable causal inference. In this review, we therefore aimed to understand how changes to the external physical environment may act to promote walking, cycling and physical activity and why these may or may not be effective. To realise this aim, the review combines evidence on the effectiveness of these interventions with an exploration of how and the extent to which the contexts — that is, the circumstances which enable or constrain behaviour change — affect outcomes reported through triggering different processes or activating different mechanisms to influence outcomes.

## 2 Methods

### 2.1 Overall approach

**Fig. 1:**
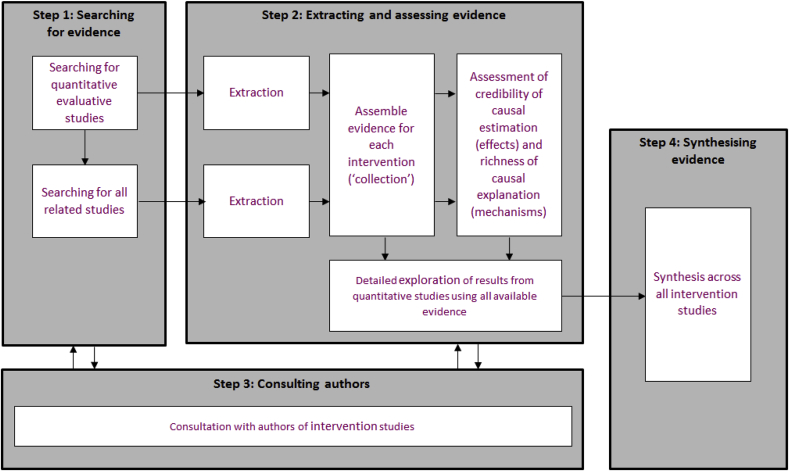
Overview of methods.

An overview of our methods is shown in Fig. 1. In the spirit of triangulating a range of types of evidence, we used principles from a range of different methods including narrative and realist reviews and qualitative analysis as recommended (Petticrew et al., 2013) and used a sequential explanatory approach (Pluye and Hong, 2014). We registered our protocol in 2016 (Panter et al., 2016).

### 2.2 Step 1: searching

#### 2.2.1 Searching for intervention studies

In a previous conceptual review (Panter et al., 2017) we identified systematic reviews of intervention studies which evaluated the impact of changes to the external physical environment on physical activity. In brief, we searched eight electronic databases (MEDLINE, Web of Science, Cochrane Reviews, ProQuest for dissertations, Health Evidence, EPPI-Centre, TRID and NICE) using terms related to reviews, physical activity, and the environment. Full details of the search terms used are shown in Additional file 1. We mined the primary studies contained within those papers to populate our sample of intervention studies. We chose this approach because investing large amounts of time in conducting new searches —in a field where new intervention studies are being published at a slow rate as demonstrated in a review from 2018 (National Institute for Health and Care Excellence, 2018) — was unlikely to be a good use of resources. We supplemented these intervention studies with papers from the review team's personal collections and contacted authors of intervention studies to identify any missing studies (see step 3).

We focussed on interventions targeting the external physical environment, defined as any change in the physical (natural) environment or the urban or constructed (built) environment that subconsciously or consciously relates to a social group or population and their walking and cycling behaviour, regardless of whether or not they have the aim of improving health. We focussed on walking and cycling behaviour to narrow the review and limit the potential number of intervention studies and we hypothesised that interventions focusing on walking and cycling would target a more limited number of potential mechanisms. It is very possible that other interventions without the aim of promoting activity may change activity indirectly, but these were not the focus. We also excluded interventions (i) which promoted or encouraged of the use of an existing environment, for example through signage as these are mainly intended to increase awareness of the environment; (ii) those that targeted additional behaviours such as public transport use and; (iii) multi-component interventions that included both environmental and individually-delivered educational components, such as information about the benefits of activity through lessons or classes, personalised travel planning or the provision of maps for walking and cycling. We excluded based on the last two criteria because few authors disentangled the effects of physical environment components from each other or from educational components. However, we acknowledge that media campaigns might be implemented alongside environmental interventions, and included studies where this was described.

We included studies conducted in adult populations, but those conducted in participants who had been prescribed exercise for a clinical condition were excluded. Studies had to assess the impact on physical activity (including as total activity or walking or cycling, assessed in free living conditions) by self-report questionnaire, objective measures, or observation of physical activity, including observation taking place in intervention areas or that involved using the intervention (such as walking or cycling along a new cycle path). We included studies that used a control or comparison group or graded measures of exposure to the intervention, and used either prospective or retrospective designs which could include randomised controlled trials, comparison trials, and quasi-experimental studies.

#### 2.2.2 Searching for related sources of evidence

From each evaluative study (referred to as the ‘intervention study’) we conducted a series of further searches to find all related sources by searching with three points of access, a well-used method in systematic searching (Booth et al., 2013; Petticrew and Roberts, 2006). To be included, sources had to describe the intervention, the individual, physical, social, political or organisational setting, or the intervention's potential causal pathway. Together the ‘intervention study’ and the ‘related sources’ comprised a ‘collection’. We first searched the reference list of each intervention study and subsequent related sources. Secondly, we conducted searches in PubMed and Web of Science for papers by the first and last author of the key intervention study. Finally, we searched for project names in PubMed and Google Scholar, where appropriate. We applied no limits on the type of source in this search and quantitative or qualitative studies, policy documents, theses, grey literature or reports were all eligible for inclusion.

### 2.3 Step 2: extraction and credibility

#### 2.3.1 Causal estimation from intervention studies

For each intervention study, two reviewers (JP and CG) independently extracted basic descriptive information on the type of intervention, the method of evaluation, the results and completed simple qualitative assessments of the credibility (representativeness, comparability, measurement, significance) using binary yes/no scores as other reviewers have done (Yang et al., 2010). We also added time between implementation and follow-up. We summed the scores for all intervention studies (possible range 0-5). For each study, we described its overall effectiveness and allocated this to one of four categories: positive significant effects; negative significant effects; inconclusive or no effects; or no assessment of statistical significance. We also assessed and extracted the evidence for specific outcomes. Further methodological details can be found in Additional file 1 and 2.

#### 2.3.2 Causal explanation for intervention studies and related sources

We drew on the principles of realist review methods and extracted evidence from all sources in each collection on contexts, mechanisms and outcomes (Pawson, 2006) using the definitions described in Table 1. Contexts, mechanisms (resources and reasoning) and outcomes could all be described at the micro or macro levels (relating to individuals, groups or areas) and act in configurations (Table 2). Breaking down these components about how change might occur implies a rather linear order, and although this may be unlikely in practice it was necessary here in order to establish gaps in the evidence to guide public health action. It also allows for the fact that one outcome might be a context for another set of configurations and that intermediate outcomes (such as use of the infrastructure) could be captured. For example, exposure to new infrastructure could lead people to begin using it for walking (outcome). As people use the infrastructure for walking (new context) they may subsequently begin to consider the possibility of using it for cycling and may take up cycling (subsequent outcome). In our assessment of the evidence, we identified whether it was based on (a) prior assumptions or theorising about how the intervention might work, (b) the author's observations tested with data, (c) the author's interpretation or (d) our interpretations as reviewers, using methods from meta-ethnography (Noblit and Hare, 1988).

**Table 1:** Definitions used.

Term	Definition
*Contexts*	‘The physical, social, political or organisational setting in which an intervention was evaluated or in which it was implemented’ (p119, Rychetnik et al., 2002)
*Mechanisms*	Those processes which described how intervention activities, and participants' interactions with them trigger change. These may be measureable or hidden (latent). In the literature there is considerable debate about how mechanisms are conceptualised and identified (Dalkin et al., 2015) and during the extraction we further separated two parts of mechanisms: the *resources* (what the intervention did) from the *reasoning* or process of change (how people or populations responded).
*Outcomes*	Those which were subject of the main evaluative study (e.g. physical activity or use of the new environment or infrastructure), or intermediate outcomes which were necessary for changes in the physical activity or use, or subsequent outcomes which followed from use or changes in physical activity

**Table 2:** Examples of resources, contexts, reasoning or processes and outcomes.

Resources are implemented …	in this context …	which leads to these changes in reasoning or a change in process	… and produces this outcome
Function the intervention performs *(e.g. segregates pedestrians from traffic or improves accessibility to destinations)*	Physical, social, political or organisational conditions in which the intervention is introduced *(e.g. flat topography, existing cycle network or socially acceptable to cycle)*	Process occurring on a group level *(e.g. gradual shifts in acceptability of cycling)*	Population level shifts in use or activity
	Physical, social or political conditions of individual exposed to the intervention (*e.g. owns a bike, able to walk, supportive of change)*	Process or reasoning *(e.g. changes in perceived safety of walking or cycling)*	Individual change

#### 2.3.3 Joint estimation causal explanation and estimation

Using an adapted version of the harvest plot method (Ogilvie et al., 2008), for each study we plotted scores for the credibility of casual estimation according to the height of the bar (scores 0-5). Studies with distal outcome measures (such as weekly time spent walking) were indicated with full-tone (black) bars, and studies with proximal or intermediate outcome measures (such as use) with half-tone (grey) bars. We annotated the bars with the credibility of the causal explanation scores (A: rich; B: thick; C: thin).

##### 2.3.3.1 Assembling evidence for each intervention

Based on the evidence presented in each collection we formed the most plausible configurations of contexts, mechanisms and outcomes and constructed a preliminary synthesis of how the intervention worked in tabular and narrative forms. We also assessed the credibility and depth of evidence about how the intervention was thought to bring about its effects using a combination of assessments of thicker and thinner descriptions (Roen et al., 2006) and conceptual richness (Ritzer, 1991) as others have done (Pearson et al., 2015). We have made some slight adaptations to these guiding criteria and described short-hand characteristics of rich, thick and thin papers. ‘Rich’ papers develop and describe underlying theoretical concepts of interventions, ‘thick’ papers contain explorations and discussions of factors affecting the implementation, whereas ‘thin’ papers provide little explanatory detail.

### 2.4 Step 3: consulting authors

For each intervention study, we identified a key contact. In some cases, we were able to identify the Principal Investigator of the study from the additional information (funding/acknowledgements) and in others we identified the lead or corresponding author as the key contact. In cases where there was doubt over the best contact, we identified two contacts. We emailed these contacts asking them to corroborate or improve our preliminary narrative synthesis of how the intervention worked, which took the form of a short paragraph or two (as described in Step 2). We also asked them to identify missing papers or unpublished material which may help describe how the intervention worked and why. We also included a list of the intervention studies and asked if they were aware of any others which we may have missed and met our criteria.

### 2.5 Step 4: synthesis across studies

We combined all of the extracted data, and two reviewers (JP and CG) synthesised the findings in three stages. First, we created a summary table which combined all context-mechanism-outcome configurations from all collections. This process served to familiarise us with the individual configurations. From this, second, we identified common functions – overarching themes – across these interventions (i.e. what the intervention actually did or provided). We then indexed all context-mechanism-outcome configurations where these functions were mentioned and retained the reference to the source and richness of the causal explanation. Third, we synthesised these combinations of contexts, mechanisms and outcomes on a more abstract level (independent of individual study details) with a focus on exploring patterns of outcomes (more successful and less successful) and on those with the strongest or most convincing evidence.

## 3 Results

### 3.1 Sources of evidence

From our initial search we identified 33 potentially relevant review papers, and 13 evaluation studies in which the interventions specifically targeted walking and cycling (Dill et al., 2014, Evenson et al., 2005, Fitzhugh et al., 2010, Fuller et al., 2013, Goodman et al., 2014, Gustat et al., 2012, Krizek et al., 2009, Mccartney et al., 2012, Merom D, 2003, Parker et al., 2013, West and Shores, 2011, West and Shores, 2015, Wilmink and Hartman, 1987) (Additional file 2, Fig. A1). Only one was a report (Wilmink and Hartman, 1987); the remainder were academic papers. Table 3 describes the nature of the interventions, the main outcomes assessed, the main results and a summary of the evidence of effectiveness. We identified studies that examined the impact of walking paths (n = 1), cycle paths or lanes (n = 5), facilities to support cycling (n = 1) and routes for walking and cycling (n = 6). Evaluative studies assessed specific proximal outcomes such as walking or cycling in the intervention area or on the route or path (n = 3) and more global outcomes such as changes in weekly levels of activity (n = 7). Three studies assessed both types of outcomes.

**Table 3:** Characteristics of and results from included studies.

Short name	Description of intervention	Main outcomes^1^	Main reported effects	Effectiveness		Explanation	
				Summary^2^	Credibility	Number of sources	Credibility^3^
Walking paths							
PACE (Gustat et al., 2012)	New walking path	Any walking for transport (SR); walking for leisure (SR); walking for transport and leisure (SR); counts of people observed in sedentary activity, walking and in MVPA	Positive effects of the intervention on numbers of people observed in MVPA. No significant effect on any of the other outcomes.	Inconclusive or no effect	4	1	Thin
Bicycle paths, lanes and routes							
DEL (Wilmink and Hartman, 1987)	New on-road and off-road cycle lanes	Share of bike trips (SR); share of car trips (SR); share of walking trips (SR)	Positive effect of the intervention on bike share, but no effects on car trips or those made on foot	Positive effect of uncertain significance	3	13	Thick
MIN (Krizek et al., 2009)	New on-road and off-road cycle lanes	Cycle mode share on the commute at the city level (SR); around specific new facilities (SR) and around major destinations (SR)	Positive effects of intervention at the city level and around specific facilities for bike mode share, but not at major destinations	Significant positive effects	3	1	Thin
NEW (Parker et al., 2013)	New cycle lane	11 h observation of cyclists on the streets and cycling with the traffic	Positive effect of the intervention for observations of cycling	Significant positive effects	2	0	Thin
PORT (Dill et al., 2014)	New cycle boulevards	Time spent in MVPA (SR); time spent cycling (SR); time spent walking (SR); any walk or cycle trip (SR); any cycle trip >10mins or walk trip >20 min (SR); number of walk trips (SR); number of cycle trips (SR)	Negative effect of intervention on minutes of cycling and number of bike trips. No effects on any of the other outcomes.	Inconclusive or no effect	2	2	Thin
SYD (Merom D, 2003)	New cycle trail	Time spent walking (SR); time spent cycling (SR); more than 150mins walking or cycling (SR); count of cyclists	Positive effects of the intervention on counts of cyclists and cycling time. No effects on walking.	Significant positive effects	4	1	Thin
Bicycle facilities							
BIXI (Fuller et al., 2013)	Bicycle hire scheme	Self-reported any cycling; any utilitarian cycling; any recreational cycling (after 1 year and 2 years separately)	Positive effects of the intervention on any cycling and recreational cycling after 2 years but not 1 year.	Significant positive effects	5	10	Thick
Routes for walking and cycling							
DUR (Evenson; KR, 2005)	Extension of an existing trail	Self-reported leisure time PA; leisure time PA in the neighbourhood; MPA; VPA; total walking, walking for transport, total cycling and cycling for transport	No effects on any outcomes	Inconclusive or no effect	3	1	Thin
GLA (Mccartney et al., 2012)	New pedestrian and cycle bridge	Count of pedestrians and cyclists per capita	Positive effects of the intervention on counts of pedestrians and cyclists	Positive effect of uncertain significance	2	0	Thin
iC (Goodman et al., 2014)	New walking and cycling routes	Self-reported total past-week walking and cycling; Total past-week physical activity (after 1 year and 2 years separately)	Positive effects of the intervention on total past-week walking and cycling and total past-week physical activity (after 2 years, no effects after 1 year)	Significant positive effects	5	12	Rich
KNOX(Fitzhugh et al., 2010)	New trail	Counts of observed PA in the neighbourhood	More people seen to be active, walking and cycling in neighbourhood.	Significant positive effects	3	3	Thin
MEC(West and Shores, 2015)	Extension of an existing greenway	Number of days spent walking; number of days spent in MPA; number of days spent in VPA.	No effect on any outcomes	Inconclusive or no effect	3	1	Thin
ROA (West and Shores, 2011)	Extension of an existing greenway	Number of days spent walking; number of days spent in MPA; number of days spent in VPA.	No effect on any outcomes	Inconclusive or no effect	3	1	Thin

^1^All primary outcomes tested are listed and where auhthors did not discriminate between primary and other outcomes all outcomes are listed; ^2^ Significant positive effects = when more than 50% of outcomes showed positive significant effects; inconclusive or no effects = when more than less than 50% of outcomes showed positive effects or when results were mixed; ^3^ Comprising a mix of intervention and evaluation theory as well as description of intervention and context. Interventions were categorised based on their main type of intervention e.g. cycle lane or facilities for cycling, although some did both eg. MIN [36]. SR: self-reported; VPA: vigorous physical activity; MVPA: moderate-to-vigorous physical activity.

In total, we identified 46 related sources (full list in Additional file 2). Each intervention had a median of 1 related source (IQR: 1-6.5) but there was significant variation in the number of related sources for each evaluative study (Table 3). The collections could be divided into four groups. The first consisted of those collections which contained numerous related sources which were predominantly academic papers (n = 2); the second contained numerous related sources which were reports and working documents (n = 1); the third contained few related sources which consisted of either one or two related policy documents, academic papers or theses (n = 8); and the fourth comprised interventions for which no related sources were identified (n = 2).

### 3.2 Summary of evidence for effectiveness of interventions

6 intervention studies reported significant positive effects, 2 reported positive effects of uncertain statistical significance and 5 reported inconsistent effects. There appeared to be no pattern of intervention effectiveness according to the type or form of intervention or the methods of data collection.

The mean credibility score was 3.4. 70% (n = 9 studies) scored 3 or less on the credibility criteria for effectiveness. Only four studies met at least four of the five criteria (Fuller et al., 2013; Goodman et al., 2014; Gustat et al., 2012, Merom D, 2003) (see Table A2, additional file 2), and of these, three reported positive effects.

### 3.3 Summary of evidence for explanation of intervention effects

We identified three common resources that interventions provide (or functions that they perform) to promote walking and cycling: (i) improving accessibility and connectivity of an area or route; (ii) improving safety from traffic and personal attack; and (iii) improving the experience of walking and cycling. Almost all studies identified improving accessibility or connectivity to destinations as the main intervention function (e.g “to make it easier for pedestrians and cyclists to reach destinations in the local area” (Ogivlie et al., 2008), and in some cases interventions changed more than one function (Goodman et al., 2014). These functions were targeted by different forms of interventions. Much of the evidence was based on thin descriptions of how interventions might bring about their effects (Table A2; Additional file 2). When we examined the nature of each piece of evidence about context or mechanism (including function, reasoning or process), we found that contexts were often described by authors and sometimes supported by data, whereas the mechanisms were rarely explicitly described and often implied, sometimes based on the authors' interpretation and most often based on the review team's interpretation.

We take each theme in turn and summarise the ways in which mechanisms were enabled (or disabled) by different (macro and micro) contexts and explore how this might have led to different outcomes. We provide a full breakdown of all studies which contributed evidence about each intervention function, context, reasoning and the outcomes (Additional file 2, Table A3). We use the short study names (as shown in Table 3) for ease of identification in the text below.

#### 3.3.1 Accessibility and connectivity

##### 3.3.1.1 Improving accessibility

Of the studies which reported positive outcomes on use of infrastructure or changes in physical activity, it appeared that both supportive and unsupportive conditions for walking and cycling could trigger increases in walking and cycling. For example, in physical contexts which were already supportive for walking and cycling, such as areas with many destinations and employment centres in easy reach and with good public transport links (BIXI), cycling might have become even more practical for short trips. For public transport users, improvements in connectivity in already supportive contexts allowed them to combine modes of transport within a single journey. Equally, in the context of a car-dominated environment (iC) or poor conditions for walking (PACE), new enabling infrastructure could encourage people towards walking and cycling as it became more convenient to walk or cycle.

For those without access to a car or those commuting, the new infrastructure might have led to cycling becoming more convenient or a viable alternative to walking and cheaper than public transport use (SYD, MIN). However, these mechanisms did not appear to have been enabled for all: for some groups such as those travelling long distances, the infrastructure might not connect with desired or essential destinations, which might explain the differential effects seen in some locations (MIN, ROA).

##### 3.3.1.2 Improving connectivity/directness

Some authors described interventions not in terms of making places more accessible but as improving the connectivity and continuity of a network or improving the directness of a route. These were reported in contexts of unsupportive conditions for walking or cycling, such as fragmented route networks (iC) or low street connectivity (PACE, KNOX). Connecting destinations appeared to make it more convenient to walk or cycle and where the infrastructure was well-used, increases in overall walking and cycling were reported (iC, KNOX).

The combination of supportive individual and environmental contexts was also reported. For existing cyclists and those living in areas with a relatively well developed cycle network, more varied and longer routes might be possible if the accessibility of an areas increases (DUR, PORT, SYD). Whilst this might lead to increased levels of cycling as people are encouraged to make more or longer trips by bike (iC), it might also reduce journey times and lead to decreases in time spent cycling (PORT, SYD) when existing trips are simply replaced by shorter routes, particularly if the effects of interventions were assessed in populations who were already quite active. This might explain the differential outcomes of some of the interventions that showed increased use of the infrastructure but decreases in walking and cycling or physical activity (for shorter routes) or no change (for trip replacement; DUR). The intervention may have been implemented in a location where there was little perceived need for new infrastructure (DUR). Residents may have perceived it as a space for recreational physical activity rather than for transport purposes, or for those who wanted connections to destinations the infrastructure did not go where it was needed (PORT).

#### 3.3.2 Traffic and personal safety

Comprehensive and connected walking and cycling networks did not simply enhance convenience or accessibility; such networks also provided the opportunity for safe routes without hazardous incursions by motor vehicles, or reduced the perceptions of crime.

##### 3.3.2.1 Segregation from motor vehicles

In the context of busy car-dominated urban environments with fast-moving traffic flows, infrastructure which segregated pedestrians and cyclists from motor vehicles appeared to alleviate concerns about traffic safety and reduce the conflict between motorists and cyclists (iC, DEL, MIN). However, this mechanism seemed difficult to achieve/trigger in some contexts. For example, where safety concerns were not fully addressed, i.e. where people may have not felt safe or comfortable on *all parts* of the journey, usage of the infrastructure remained low (SYD). For example, users might still have to cross busy roads to reach new infrastructure and the off-road path might have reduced the conflict in some parts of a journey but not all (SYD), and this might be particularly important for those who do not already walk or cycle (PORT). Supportive conditions also appeared to facilitate usage of infrastructure, but this may have resulted in trip replacement rather than encouraging new walking or cycling trips. For example, for existing cyclists or in areas where there were existing on-road cycle paths, segregation provided a safer place to cycle away from traffic (DUR, GLA). In this case, infrastructure might change *where* cycling occurs rather than the duration or frequency of cycling (GLA).

##### 3.3.2.2 Reducing perceptions of crime

New walking and cycling paths may have alleviated concerns about personal safety and the risk of attack by providing well-lit places for walking and cycling and subsequently encouraged the use of new infrastructure (iC). However, if these spaces or routes themselves were used as a location for criminal activities, then the perception of risk may have increased and this might explain why the usage of infrastructure was low. The provision of a hire bike scheme might also reduce the perception of crime; less desirable hire bikes might be less of an object for theft than personal bikes and reduce any concerns about personal property loss (BIXI). In contexts where the fear of bike theft is high this may encourage the use of bike sharing schemes; however, if only existing cyclists use the hire bikes, bike hire schemes might not change population levels of time spent cycling.

#### 3.3.3 Other attributes of quality of the experience

Finally, authors suggested that interventions worked to promote physical activity by making areas more aesthetically pleasing. They were mostly describing greenways for walking or cycling, which might be more likely to attract users who were undertaking recreational activity. This included interventions in dense urban areas or where there were poor street conditions for walking (such as uneven or cracked pavements); interventions made areas or routes more aesthetically pleasing and this might have provide residents with a more pleasant place for recreational walking (which may lead to more walking) (KNOX) and encourage more people to be active in the neighbourhood (NEW, PACE). Other authors suggested that, just as for safety, for those who already walked or cycled, newly beautified routes and areas might change where walking or cycling takes place but it cannot be assumed that this would lead to increases in walking or cycling. In one study (DEL), authors suggested interventions might make cycling more comfortable and lead to a smoother ride and greater comfort and enjoyment of cycling. This might encourage uptake of cycling not (only) for leisure, but for those making commuting or business trips.

### 3.4 Joint assessment of effectiveness and explanations

**Fig. 2:**
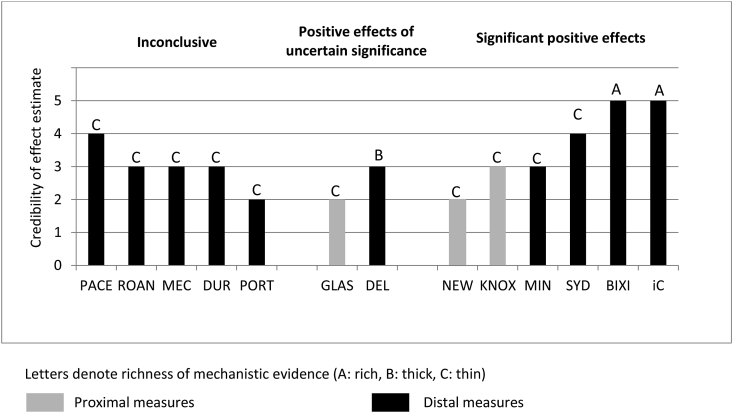
Evidence for intervention effects and explanation.

Fig. 2 shows the evidence for intervention effects and explanation for each study. The distribution of the tones, heights and annotations of the bars suggests that the evidence was mostly contributed by studies with low credibility of causal estimation, some of which found evidence on proximal outcomes (such as use) rather than most distal outcomes. Studies reporting positive effects comprised a mix of higher credibility of causal estimation and explanation, but in general studies with higher scores of credibility of causal estimation were more likely to find evidence of significant positive effects. Studies which had low scores on credibility of estimation also tended to be those which also scored lower on explanation.

### 3.5 Generalisable context and mechanism interactions

**Fig. 3:**
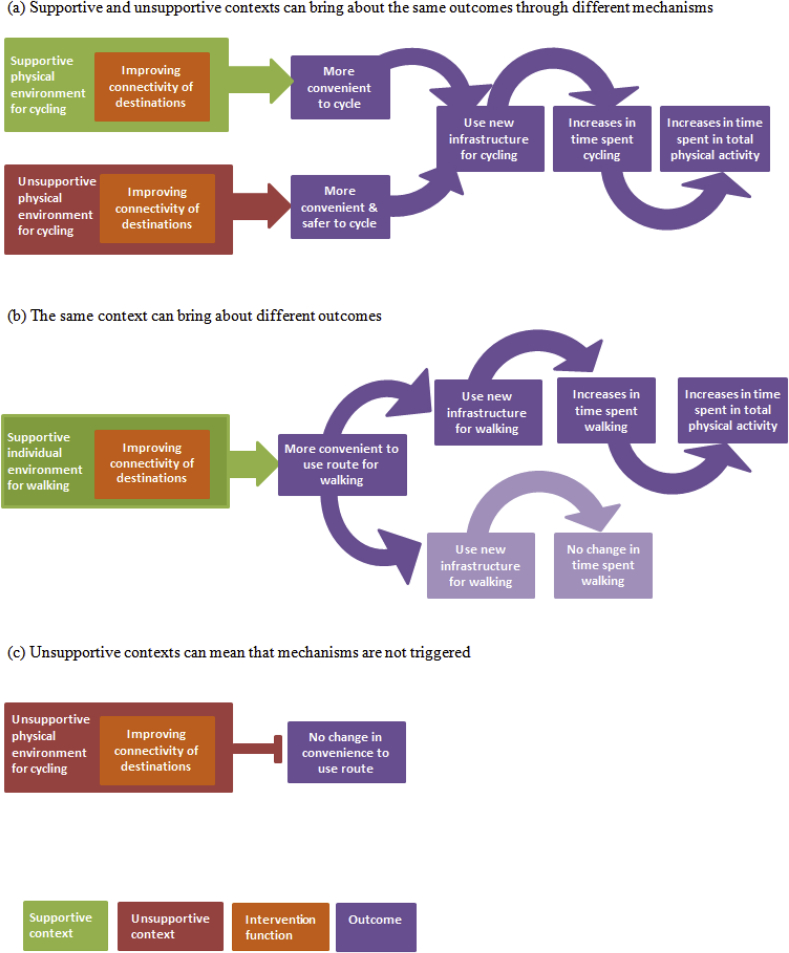
Generalisable configurations of contexts, mechanisms and outcomes.

From the evidence reviewed we distilled three potential ways in which the interaction of an intervention's function with different contexts may lead to processes and outcomes being enabled or disabled, as shown in Fig. 3. In the example, we have consistently used the connectivity as a function of the intervention. This provides a clear illustration of the different interactions with contexts, mechanisms and resulting outcomes but this could be easily applied to any of the other functions or mechanisms.

Firstly, different (supportive or unsupportive) contexts may bring about the same outcomes through different mechanisms (panel a). For example, in the context of a supportive environment, such as a densely populated area with destinations and good public transport links, increased connectivity between destinations may mean that cycling becomes more convenient and people switch from the car to cycling, leading to increases in the number of cycling trips and time spent cycling (top row). In the context of an unsupportive environment, such as an area with a fragmented cycle network, new infrastructure may also make it more convenient to cycle than to use a car, resulting in similar overall increases in the number of cycling trips and time spent cycling. Equally, in an unsupportive context characterised by a fragmented cycle network and busy traffic, new infrastructure may make it not only more convenient but also safer to cycle (bottom row). This highlights the multiple functions that an intervention may provide. The implementation of an intervention in an unsupportive context may alter people's choices by ‘tipping the balance’ in favour of walking or cycling.

Secondly, a single context (either supportive or unsupportive) may trigger the same mechanisms but bring about different outcomes depending on how interventions are used and either amplify or dampen expected effects (panel b). For example, the new infrastructure may improve the connectivity of an area, making it more convenient to walk to new destinations previously out of reach. On the one hand, people may use the new infrastructure for new walking trips, leading to increases in overall walking and consequently increases in total physical activity (top row). On the other hand, those who already walk may use the new infrastructure to reach their existing destinations. This would constitute a change in routes (i.e. spatial displacement of existing walking trips) rather than the uptake of walking for new trips, and may therefore not lead to an increase in the overall quantity of walking (bottom row).

Thirdly, environmental changes occurring in unsupportive physical contexts may not trigger anticipated mechanisms (panel c). For example, in the context of busy traffic, improvements in the connectivity may be insufficient to trigger change if busy traffic represents a more substantial barrier to cycling. This may mean that environments are not used as much as expected, or not used by those who could benefit the most, and changes in behaviour may therefore not be observed.

## 4 Discussion

### 4.1 Summary of findings

We focussed our review on evaluative studies of changes to the physical environment targeting walking and cycling and found studies varying in scale and credibility. We identified heterogeneous interventions ranging from small-scale to whole-network improvements which performed multiple functions and altered specific characteristics of the environment (e.g. changing the accessibility of destinations within an area, or segregating pedestrians and cyclists from motor vehicles). We identified some evidence that individual or population levels of walking and cycling and the supportiveness of the physical and wider social environment were important contexts. However, there was little information about potential mechanisms. The most plausible mechanisms concerned (i) improving accessibility and convenience of walking and cycling and (ii) reducing potential conflict between users.

### 4.2 Strengths and constraints

A key strength of our review is that we combined evidence about the effectiveness of environmental interventions (‘causal estimation’) and how the effects may be brought about (‘causal explanation’) and assessed the credibility of that evidence. To do this, we based our synthesis on the function rather than the form of the interventions, in contrast to many previous reviews on the topic (Mayne et al., 2015; Smith et al., 2017; Stappers et al., 2018). This required methods from a range of disciplines to be developed and adapted, and the combination of complementary qualitative and quantitative evidence. It allowed us to draw together evidence about different surface forms of environmental change which work in similar ways. By triangulating different types of evidence we were able to make steps towards more generalisable causal inferences based on evidence from specific cases, which has been described as developing an understanding of “the universal” that can be drawn from intense understanding of “the particular”(Hawe, 2015).

We focussed the review on environmental changes that targeted walking and cycling specifically. In doing so, we have demonstrated a method which could be applied (with some adaptations) to interventions which target physical activity more generally or other health outcomes. We have no reason to believe that these mechanisms would not apply to activity more generally. After piloting qualitative comparative analysis (QCA) in a sample of studies (Thomas et al., 2014), we found it difficult to apply binary classifications of conditions and contexts to each study, and therefore used principles of this method to make comparisons between studies without quantification. Given the difficulties of conducting studies using matched control and intervention groups we included studies using any form of controlled comparisons, as these are likely to provide the best available evidence of intervention effects in this field (Humphreys et al., 2017).

We may have missed some relevant recent evaluations as we only used published systematic reviews, and even those published up to 2017 constrained their search to those studies published in 2016. However, given the slow rate of publication of studies in this area (National Institute for Health and Care Excellence, 2018) and the ‘demonstration’ nature of the review we believe this pragmatic approach is appropriate. We also contacted authors to identify any missing papers and to ensure our interpretations were valid and grounded in the evidence. In addition, our approach to identifying related evidence was restricted to sources related to the specific intervention under consideration. It would have been possible to expand our search and examine sources of evidence which describe the relationships between changes in environmental perceptions and physical activity behaviours in observational studies without evaluating an exogenous intervention (Jongeneel-Grimen et al., 2014). This would have substantially increased the number of sources for evidence synthesis and was outside the scope of this review. However, a previous review using qualitative observational evidence in deprived contexts identified similar mechanisms (safety, accessibility and comfort) (Kramer et al., 2017), suggesting that a broader range of sources might not have yielded a substantially different set of mechanisms. Future reviews could include a broader set of evidence.

### 4.3 Limitations of previous reviews

Although several previous reviews have concluded that new infrastructure may be effective in increasing walking or cycling (Mayne et al., 2015; Smith et al., 2017; Stappers et al., 2018) others report a more mixed patterns of results (National Institute for Health and Care Excellence, 2018). All authors report challenges in summarising and synthesising evidence of effects because there were relatively few studies and they were heterogeneous in terms of context, study design and quality. Few reviews have more deeply considered the contexts or mechanisms of interventions. A previous realist review examined a specific programme of voluntary resettlement (Jackson et al., 2009), whilst others focussed on a specific context (deprived areas (Kramer et al., 2017)) or population group (older adults (Yen et al., 2014)). Both of these latter reviews were limited in different ways. In the case of the review in deprived areas, the results concerned the evidence for different mechanisms operating in a single context, not on how *variations in context* might trigger different mechanisms. In the review in older adults, the authors were unable draw conclusions on the contextual influences and causal mechanisms because of a limited evidence base and so were only able to describe influences on older adults’ mobility. By using intervention studies as a lens for this review, we were able to draw conclusions about the role of context in the success of interventions.

### 4.4 Implications for causal estimation

Some intervention studies reported methodological challenges which might have explained the lack of success of interventions. These included delays in intervention implementation (Dill et al., 2014), short time to follow-up (a result of delays in some cases, and a consequence of study design(Parker et al., 2013) in others) and insufficient dose of the intervention (Dill et al., 2014; Evenson et al., 2005). In reviewing these studies we also noted that many of the outcomes measured were not specific to the intervention and might have been too coarse to detect effects (e.g. number of days spent walking; (West and Shores, 2011; West and Shores, 2015);). Some found effects for some cycling but not walking and this might have been because the use of the infrastructure for walking was limited and thus there was less power to detect effects (Merom et al., 2003). A mix of proximate outcomes (such as use of the intervention) and more distal ones (such as weekly time spent in cycling) may be useful in explaining the success or otherwise of interventions.

70% (n = 9 studies) scored 3 or less on the credibility criteria for effectiveness and relatively few studies scored highly (4/13 scoring at least 4 out of 5). Acknowledging the challenges of conducting such studies, the interdisciplinary nature of the research, and that different study designs are appropriate and can provide robust information (Humphreys et al., 2017), we used criteria that were broad and inclusive. None of the studies were judged to perfectly meet the criteria. All studies had limitations which are inherent in this complex and challenging area of research, yet we can be more confident about some studies than others (Humphreys et al., 2017). On one hand some did not take in consideration any socioeconomic and geographical differences between control and intervention groups (e.g. (West and Shores, 2011)). On the other hand, others made attempts to provide information about the representativeness of the sample and the comparability of their samples (e.g (Fuller et al., 2013)).

### 4.5 Implications for causal explanation

Despite repeated calls for greater *a priori* theoretical consideration of how interventions might work (Panter and Ogilvie, 2015; Stappers et al., 2018; Petticrew, 2015), the evidence base for the mechanisms and context of interventions was limited. Although context was mentioned this was often superficial, with little discussion of *how* the interaction between context and intervention might have resulted in the observed effects. Similarly, where mechanisms were described these were related to intervention functions (such as improved accessibility or connectivity). There was little evidence for reasoning or processes and at best this could be described as interpretation and in other cases this was speculation. Some sources articulated specific intervention hypotheses to guide the analysis and mediation analysis to quantitatively investigate mechanisms, but again this was rare. The more credible ‘outcome’ studies were more likely than the less credible studies to report positive outcomes and to present credible investigation of mechanisms. Quantitative studies from two collections (iC and BIXI) found some evidence that exposure to new infrastructure was accompanied by changes in individually orientated psychosocial mediators, such as intention to use new infrastructure, or environmental mediators such as perceptions of safety. However, these mediators often explained a limited amount of intervention effects. It is possible that alternative mechanisms such as modelling or other unspecified mechanisms might be operating.

We found some evidence that interventions were considered with the wider physical and social system in policy documents and qualitative or mixed method studies. These sources of evidence are traditionally viewed as lower quality (Ogilvie et al., 2005), and although they were few in number here, we found that they were useful in painting a conceptually richer picture of potential contexts and mechanisms. These sources often embraced a systems thinking approach and reflected on how interventions change relationships, displace activities, and redistribute material, social, cultural and physical resources (Hawe, 2015). Environmental interventions such as those designed to promote walking and cycling – by their very nature – couple with and embed within the physical and social context. This coupling inevitably prompts changes in the social environment and changes relationships between groups (e.g. where conflict is reduced or prioritisation is given to some users and taken from others). Greater consideration of networks of person-time-place interactions using a variety of different types of data, including qualitative and mixed methods, may offer one way to provide more insight into the combinations of context and mechanisms in order to understand how interventions work (Panter et al., 2017). Guidance for public health evaluations suggests practical ways in which context can be taken into account using both qualitative and quantitative methods (Craig et al., 2018). Understanding how interventions work provide key signposts about the potential generalisability of research, which increases the utility of existing evidence for informing policy and reducing waste in research.

## 5 Conclusions

We have shown that it is feasible to synthesise evidence concerning environmental interventions to promote walking and cycling and apply an inductive and narrative method to produce policy-relevant findings about how these interventions may work. 13 studies evaluated interventions targeting walking and cycling and only 4/13 studies scored 4 or more on the credibility criteria for effectiveness. Higher quality studies were more likely to report positive effects. Only two studies provided conceptually rich evidence of mechanisms. We identified three common resources that interventions provide: (i) improving accessibility and connectivity; (ii) improving traffic and personal safety; and (iii) improving the experience of walking and cycling. The most effective interventions appeared to target accessibility and safety in supportive and unsupportive individual and physical contexts. In general, studies provided some information on contexts but little information about potential mechanisms. Evidence about contexts and mechanisms can help to understand the generalisability and transferability of interventions and their effects. Future evaluative research should consider contexts and mechanisms to distil more generalisable ways in which interventions work. This will ensure the findings are useful to policymakers.

## Funding

JP and DO receive support from the Medical Research Council [Unit Programme number U106179474]. This work was undertaken by JP in the course of an NIHR fellowship (PDF-2012-05-157) and within the Centre for Diet and Activity Research (CEDAR), a UKCRC Public Health Research Centre of Excellence. Funding for CEDAR from the British Heart Foundation, Cancer Research UK, Economic and Social Research Council, Medical Research Council, National Institute for Health Research, and the Wellcome Trust, under the auspices of the UK Clinical Research Collaboration, is gratefully acknowledged. The CEDAR grant is managed by the Medical Research Council (grant code MR/K023187/1). The funders had no role in study design, data collection and analysis, decision to publish, or preparation of the manuscript.

## Author contributions

JP, CG and DO designed the search strategy, which was executed by JP. JP and CG screened the initial results of the literature searches and extracted data and all authors appraised and analysed the findings. JP drafted the manuscript. All authors contributed to the critical revision of the manuscript and approved the final version.

## Conflicts of interest

We have no competing financial interests. We have conducted evaluations in this field and one of the authors of this review also led the evaluation of iConnect

## Appendix A Supplementary data

Revised_SR May 19_page no 27 to 36 Revised_SR May 19_page no 27 to 36
