# Supplementary material for: Title: Can changing the physical environment promote walking and cycling? A systematic review of what works and how
Source: Health Place. 2019 Jul;58:102161. doi: 10.1016/j.healthplace.2019.102161 (PMC6737987; doi:10.1016/j.healthplace.2019.102161)
Supplement: Revised_SR May 19_page no 27 to 36 [file mmc1.docx]

**Additional file 1: Additional methodological details**

**Table A1:** Search terms used to identify systematic reviews

| **Study design** | **Physical activity** | **Environment** |
| --- | --- | --- |
| concept* OR theor* OR  framework OR review* OR  systematic OR synthesis OR summary | physical activity OR exercise OR walking OR bicycling OR cycling | environ* |

**Allocating summary of effectiveness for each study**

We documented the effects of the intervention on each outcome investigated. Many studies tested multiple outcomes so we also determined the overall of evidence of effectiveness for each study. Some studies presented conflicting outcome data (e.g. an effect for self-reported measures of walking or cycling, but not overall physical activity; or an effect on observed numbers of people who were physically active, but no effect on self-reported levels of total physical activity). In such cases, reviewers appraising each study had to reach an agreed overall judgement about how the results should be interpreted. We identified all primary outcomes and where authors did not discriminate, all tested outcomes are listed. We judged studies as providing evidence of significant positive effects when more than 50% of outcomes showed positive significant effects, significant negative effects when more than 50% of outcomes showed negative significant effects and inconclusive or no effects when less than 50% of outcomes showed positive effects or when results were mixed. When no assessment of statistical significance was noted, we categorised these separately.

**Additional file 2: Additional results**

Figure A1: Flow chart of studies through the review


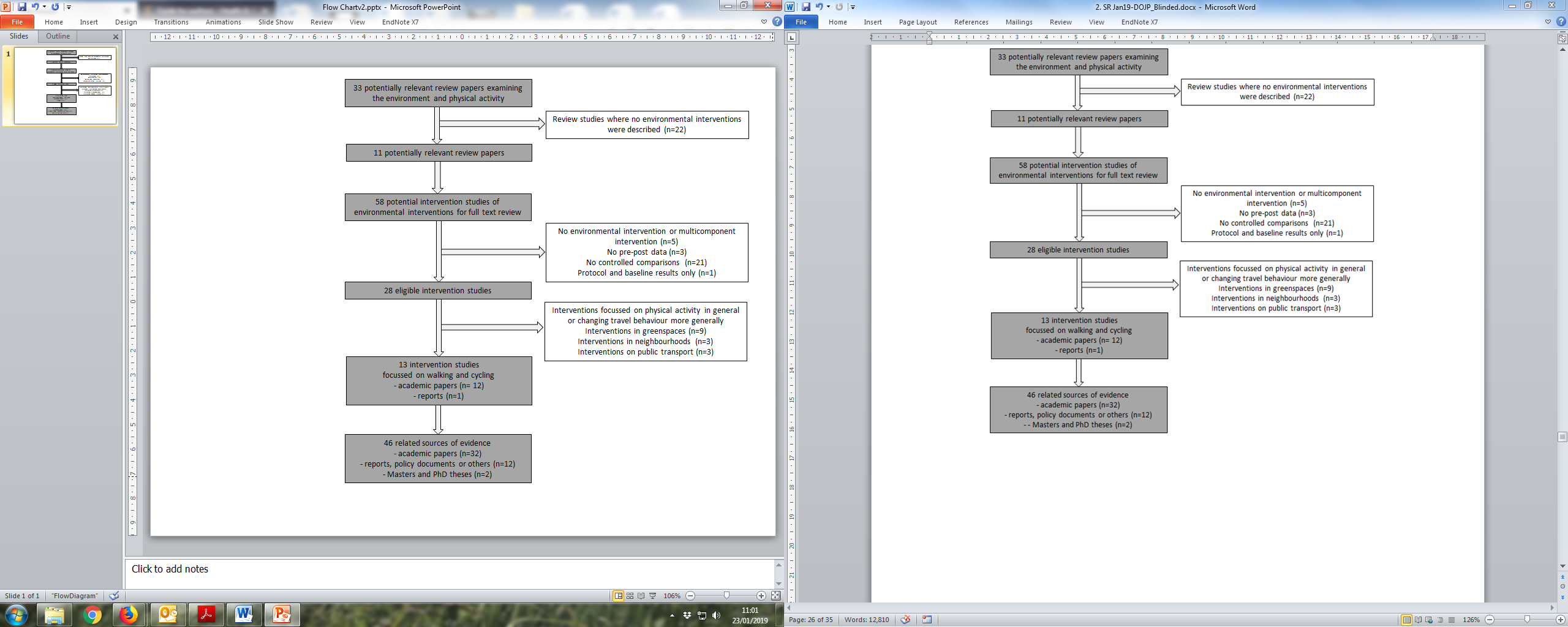
**Full reference list for all related sources**

1. Action for Bikes, Bike Plan New South Wales. Roads and Traffic Authority, 1999.

2. Adams EJ, Goad M, Sahlqvist S, Bull FC, Cooper AR, Ogilvie D: Reliability and validity of the transport and physical activity questionnaire (TPAQ) for assessing physical activity behaviour. PLoS One 2014, 9:e107039.

3. Adams EJ, Goodman A, Sahlqvist S, Bull FC, Ogilvie D: Correlates of walking and cycling for transport and recreation: factor structure, reliability and behavioural associations of the perceptions of the environment in the neighbourhood scale (PENS). Int J Behav Nutr Phys Act 2013, 10:87.

4. Andersen L, Gustat J, Becker AB: The Relationship Between the Social Environment and Lifestyle-Related Physical Activity in a Low-Income African American Inner-City Southern Neighborhood. Journal of Community Health 2015, 40:967-974.

5. Belanger-Gravel A, Gauvin L, Fuller D, Drouin L: Association of implementation of a public bicycle share program with intention and self-efficacy: The moderating role of socioeconomic status. J Health Psychol 2014.

6. Belanger-Gravel A, Gauvin L, Fuller D, Drouin L: Implementing a Public Bicycle Share Program: Impact on Perceptions and Support for Public Policies for Active Transportation. J Phys Act Health 2015, 12:477-482.

7. Bernatchez AC, Gauvin L, Fuller D, Dubé AS, Drouin L: Knowing about a public bicycle share program in Montreal, Canada: Are diffusion of innovation and proximity enough for equitable awareness? J Transp Health 2015, 2:360-368.

8. Bovy P, Den Adel D: Evaluatie Fietsroutenetwerk Delft : Mobiliteit in De Middelgrote Steden. (Evaluation Study Delft Bicycle Network : Travel Pattern in Medium-Sized Cities.). 1987.

9. Bovy PHL: Changes in Traffic Intensity as a Result of the Delft Bicycle Route Plan. (Wijzigingen Verkeersintensiteiten Door Delfts Fietsrouteplan I.). Verkeerskunde 1988, 39:p54-58.

10. Bovy PHL, Veeke P: Changes in Bicycle and Car Traffic Volume after Implementation of the Delft Bicycle Network Enhancement Plan. (Intensiteitspatronen Voor En Na Realisering Van Het Delftse Fietsrouteplan.). Bijdragen Verkeerskundige Werkdagen 1987, 29.

11. Brand C, Goodman A, Ogilvie D: Evaluating the impacts of new walking and cycling infrastructure on carbon dioxide emissions from motorized travel: a controlled longitudinal study. Appl Energy 2014, 128:284-295.

12. Brand C, Goodman A, Rutter H, Song Y, Ogilvie D: Associations of individual, household and environmental characteristics with carbon dioxide emissions from motorised passenger travel. Appl Energy 2013, 104:158-169.

13. Burchfield RA, Fitzhugh EC, Bassett DR: The Association of Trail Use With Weather-Related Factors on an Urban Greenway. J Phys Act Health 2012, 9:188-197.

14. Cartier Y, Benmarhnia T, Brousselle A: Tool for assessing health and equity impacts of interventions modifying air quality in urban environments. Evaluation and Program Planning 2015, 53:1-9.

15. Dill J, Broach J: Travel to Common Destinations An Exploration Using Multiday GPS Data. Transportation Research Record 2014:84-91.

16. Fuller D: Potential of built environment interventions involving deployment of public bicycles to increase utilitarian cycling: the case of BIXI in Montreal, Quebec. Université de Montréal, Département de médecine sociale et préventive; 2012.[PhD thesis]

17. Fuller D, Gauvin L, Kestens Y: Individual- and area-level disparities in access to the road network, subway system and a public bicycle share program on the Island of Montreal, Canada. Ann Behav Med 2013, 45 Suppl 1:S95-100.

18. Fuller D, Gauvin L, Kestens Y, Daniel M, Fournier M, Morency P, Drouin L: Use of a new public bicycle share program in Montreal, Canada. Am J Prev Med 2011, 41:80-83.

19. Fuller D, Gauvin L, Kestens Y, Morency P, Drouin L: The potential modal shift and health benefits of implementing a public bicycle share program in Montreal, Canada. Int J Behav Nutr Phys Act 2013, 10:66.

20. Fuller D, Gauvin L, Morency P, Kestens Y, Drouin L: The impact of implementing a public bicycle share program on the likelihood of collisions and near misses in Montreal, Canada. Prev Med 2013, 57:920-924.

21. Goodman A, Sahlqvist S, Ogilvie D: Who uses new walking and cycling infrastructure and how? Longitudinal results from the UK iConnect study. Prev Med 2013, 57:518-524.

22. Gustat J, Rice J, Parker KM, Becker AB, Farley TA: Effect of changes to the neighborhood built environment on physical activity in a low-income African American neighborhood. Preventing chronic disease 2012, 9:E57.

23. Hartman J: The Delft bicycle network. In The greening of urban transport: planning for walking and cycling in western cities. Edited by Tolley J. London: Belhaven Press; 1990

24. Hartman J: The Delft bicycle network revisited. In The greening of urban transport: planning for walking and cycling in European cities. Edited by Tolley R: Wiley; 1997

25. Katteler H, Förg O, Brög W: Evaluatie Fietsroutenetwerk Delft: Het Verplaatsingsgedrag: vooronderzoek. (Evaluation Bicycle Network Delft: The Travel Behaviour: before study.). Nijmegen: Instituut voor toegepaste sociologie; 1984.

26. Krizek KJ, Barnes G, Thompson K: Analyzing the effect of bicycle facilities on commute mode share over time. J Urban Plann Dev 2009, 135:66-73.

27. Louisse CJ, Ten Grotenhuis DH, Van Vliet JMC: Evaluation of the Bicycle Network of Delft: Lessons for Integral Urban Traffic Policy. (Evaluatie Fietsroutenetwerk Delft: Lessen En Leergeld Voor Integraal Stedelijk Verkeersbeleid.). Colloquium Vervoersplanologisch Speurwerk 1994, 56.

28. Ma L, Dill J: A37 Does The Installation of Bicycle Boulevards Improve Residents’ Perceptions of The Bicycling and Walking Environment? A Panel Study. Journal of Transport & Health 2015, 2:S24.

29. Mecklenberg Valley Greenways: Nature Preserves and Greenways Mecklenberg County Park and Recreation. Mecklenberg, North Carolina, USA; 2008.

30. Ministry of Transport and Public Works: Evaluation of the Delft bicycle network: summary report of the before study. The Hague: Ministry of Public Works; 1986.

31. Ogilvie D, Bull F, Cooper A, Rutter H, Adams E, Brand C, Ghali K, Jones T, Mutrie N, Powell J, et al: Evaluating the travel, physical activity and carbon impacts of a 'natural experiment' in the provision of new walking and cycling infrastructure: methods for the core module of the iConnect study. BMJ Open 2012, 2:e000694.

32. Ogilvie D, Bull F, Powell J, Cooper AR, Brand C, Mutrie N, Preston J, Rutter H: An applied ecological framework for evaluating infrastructure to promote walking and cycling: the iConnect study. Am J Public Health 2011, 101:473-481.

33. Panter J, Ogilvie D: Theorising and testing environmental pathways to behaviour change: natural experimental study of the perception and use of new infrastructure to promote walking and cycling in local communities. BMJ Open 2015, 5.

34. Parker KM, Gustat J, Rice JC: Installation of bicycle lanes and increased ridership in an urban, mixed-income setting in New Orleans, Louisiana. J Phys Act Health 2011, 8 Suppl 1:S98-S102.

35. Roanoke Valley Greenways: Roanoke Valley Greenways Annual Report 2007-2008. Roanoke, USA; 2008.

36. Sahlqvist S, Goodman A, Cooper AR, Ogilvie D: Change in active travel and changes in recreational and total physical activity in adults: longitudinal findings from the iConnect study. Int J Behav Nutr Phys Act 2013, 10:28.

37. Sahlqvist S, Goodman A, Jones T, Powell J, Song Y, Ogilvie D: Mechanisms underpinning use of new walking and cycling infrastructure in different contexts: mixed-method analysis. Int J Behav Nutr Phys Act 2015, 12:24.

38. Sahlqvist S, Song Y, Bull F, Adams E, Preston J, Ogilvie D: Effect of questionnaire length, personalisation and reminder type on response rate to a complex postal survey: randomised controlled trial. BMC Med Res Methodol 2011, 11:62.

39. Sahlqvist S, Song Y, Ogilvie D: Is active travel associated with greater physical activity? The contribution of commuting and non-commuting active travel to total physical activity in adults. Prev Med 2012, 55:206-211.

40. Song Y, Preston J, Ogilvie D: New walking and cycling infrastructure and modal shift in the UK: A quasi-experimental panel study. Transportation Research Part A: Policy and Practice 2017, 95:320-333.

41. Song Y, Preston JM, Brand C: What Explains Active Travel Behaviour? Evidence from Case Studies in the UK. Environment and Planning A 2013, 45:2980-2998.

42. Tolley R: Calming traffic in residential areas. Tregaron, Dyfed: Brefi Press; 1990.

43. Vallar J-P, Kerveillan A: Politiques en faveur du développement du vélo: bonnes pratiques de villes européennes. ADEME/Energie-Cités; 2001.

44. Walker JG, Evenson KR, Davis WJ, Bors P, Rodriguez DA: A Tale of Two Trails: Exploring Different Paths to Success. J Phys Act Health 2011, 8:523-533.

45. Wolff D, Fitzhugh EC: The Relationships between Weather-Related Factors and Daily Outdoor Physical Activity Counts on an Urban Greenway. Int J Environ Res Public Health 2011, 8:579-589.

46. Wolff-Hughes DL, Fitzhugh EC, Bassett DR, Cherry CR: Greenway Siting and Design: Relationships With Physical Activity Behaviors and User Characteristics. J Phys Act Health 2014, 11:1105-1110.

**Table A1:** Summary of credibility of intervention studies

| **Short name** | **Intervention** | **Representativeness^1^** | **Comparability ^2^** | **Measurement^3^** | **Significance^4^** | **Time^5^** | **N criteria met** |
| --- | --- | --- | --- | --- | --- | --- | --- |
| **POR** | Bicycle boulevards | No | No | Yes | Yes | No | 2 |
| **GLA** | New pedestrian & cycle bridge | No | No | Yes | No | Yes | 2 |
| **NEW** | New walking path | No | No | Yes | Yes | No | 2 |
| **ROA** | Extension of an existing greenway | No | No | No | Yes | Yes | 2 |
| **DUR** | Extension of an existing trail | No | Yes | Yes | Yes | No | 3 |
| **KNO** | New trail | No | No | Yes | Yes | Yes | 3 |
| **DEL** | Network improvements | Yes | Yes | Yes | No | N/A | 3 |
| **MIN** | New bike facilities | Yes | Yes | N/A | Yes | N/A | 3 |
| **MEC** | Extension of an existing greenway | No | Yes | No | Yes | Yes | 3 |
| **PACE** | New walking infrastructure | No | Yes | Yes | Yes | Yes | 4 |
| **SYD** | New bike trail | Yes | Yes | Yes | Yes | No | 4 |
| **BIXI** | Bike hire scheme | Yes | Yes | Yes | Yes | Yes | 5 |
| **iC** | New walking and cycling routes | Yes | Yes | Yes | Yes | Yes | 5 |

N/A Not specified

1 Were study samples randomly recruited from the study population with a response rate of at least 65% or were they representative of the study population (or weighted to ensure comparability)?

2 Were baseline characteristics of intervention and control groups, populations, or areas comparable, or, if there were important differences in potential confounders were they appropriately adjusted for in analysis?

3 Were the instruments used to measure behaviour either shown to be valid and reliable in published research or in a pilot study, or otherwise recognised as an established method? (We assume direct observation is an established method)

4 Was a test of statistical significance applied specifically to the observed net change in behaviours?

5 Is there sufficient time (at least 6 months) between implementation and follow-up?

**Table A2:** Conceptual richness of collections

| **Rich^1^** | **Thick but not Rich ^1,2^** | **Thin ^1,2^** |
| --- | --- | --- |
| Theoretical concepts are unambiguous and described in sufficient depth to be useful | More detailed description of what the intervention did and how it might work | Insufficient information on intervention to enable a description of the potential way in which it might work to be surfaced (relatively a-theoretic description of intervention) |
| Concepts sufficiently developed and defined to enable understanding *without* the reader needing to have first-hand experience of an area of practice | Recognition and consideration of the factors affecting the implementation and the context within which the intervention was being evaluated (e.g. implemented in a supportive environment for walking or cycling) | Limited comments on, or discussion of, the ways in which factors associated with the intervention may have influenced the reported results (description of context or how it worked) |
| Concepts grounded strongly in a cited body of literature | Recognition and discussion of the strengths and weaknesses of the intervention as implemented | Limited or no discussion of the strengths and weaknesses of the intervention as implemented |
|  | Some attempt to explain anomalous results and findings with reference to the underlying conceptual ways it might work, context and data,(e.g. expected an effect on utilitarian walking found an effect on leisure time and explored potential reasons) | No attempt to explain anomalous results and findings with reference to context and data |
|  | Intervention theory checking: interrogation of possible explanations for results or mechanisms of intervention (e.g. how deep have authors thought about what went on?) | No intervention theory checking or testing |
| **Studies** |  |  |
| **iC** (*a priori* conceptual framework, explored and tested a variety of paths and mechanisms within the framework, through mixed and multiple methods and data; also thick description /interrogation of possible explanations, limitations/weakness of intervention and contextual variation) | **BIXI** (papers testing particular parts of the causal pathway but with narrow view of how it might work; testing of mediators and subsequent outcomes) | **DUR** (limited description of context and factors affecting the intervention (extension of existing infrastructure, high general pre-use), but limited discussion of anomalous findings; authors had collected lots of data, but appears they haven’t analysed it in a way to explain mechanisms) |
|  | **DEL** (mixed-method; initial exploratory work of potential mechanisms – convenience over safety – some testing and explanatory discussion e.g. on mental maps; description of theory of change and investigation of several subsequent outcomes) | **PACE** (limited description of context – people walk and use urban spaces such as sidewalks for exercise – and mechanisms why intervention worked; some indication that residents perhaps reliant on walking or pt but not discussed) |
|  | **PORT** (some description of context and of, and testing of, potential mechanisms such as neighbourhood attractiveness and safety; discussion of limitations of time frame/measurement, variations and limits in implementation; attempt to explains some unexpected findings) | **KNOX** (generally thin and unclear description of contexts and of mechanisms) |
|  |  | **MIN** (limited description of context and mechanisms – although some discussed and tests like route choice and that going out of their way to use cycle paths might indicate need for safe routes) |
|  |  | **SYD** (limited description of context, and mixed-method exploration and testing of mechanisms around messaging and location, and discussion of additional factors such as trail quality) |
|  |  | **GLA** (some description of context and limited discussion of mechanisms – geographical variation and SES) |
|  |  | **NEW** (limited description of context but some discussion of potential mechanisms (gender, messaging, modelling, use of lane vs road) |
|  |  | **ROA** (very limited description of context or mechanisms) |
|  |  | **MEC** (Limited discussion of context and no attempt to explain anomalous results and findings with reference to context and data, only study design) |

^1^**Roen et al. 2006 ^2^ Pearson et al. 2015**

**Table A3:** Explanations for more or less successful interventions

|  |  | **Potential explanations for success** | | **Potential explanations for lack of (less) success (than expected)** | |
| --- | --- | --- | --- | --- | --- |
|  |  | Conditions in the environment or of/in the population | Potential mechanisms | Conditions in the environment or of/in the population | Potential mechanisms |
| **(i) ACCESSIBILITY** | |  |  |  |  |
| **Provides access to places or facilities for PA** | |  |  |  |  |
| iC | Significant positive effects | Car dominated environment  In pleasant surroundings | More convenient to walk or cycle  Improved access to recreational walking routes may mean it becomes more pleasant to walk which explains use for walking | Space is used as location for crime or antisocial behaviour | May generate concerns about personal safety which could lead to low usage |
| BIXI | Significant positive effects | In population dense area with destinations and good public transport links | Cycling becomes practical for short trips (and connects cycling and public transport use) and encourages use | Those with lower levels of education or poorer health | May have no desire to know /seek information and no intention to change mode to BIXI |
| PACE | Inconclusive or no effect | Poor conditions for walking | More convenient to walk to destinations |  |  |
| SYD | Significant positive effects | Fragmented network with busy traffic  Those without access to a car and making long trips | More convenient to cycle which encourages use and awareness  Cycling becomes a viable alternative to walking and cheaper than public transport which may lead to increases in time spent cycling |  |  |
| ROA | Inconclusive or no effect |  |  | Fragmented network with busy traffic | Infrastructure doesn’t meet need; it’s too far away |
| GLA | Positive effect of uncertain significance |  |  | Existing cyclists | Infrastructure may change where cycling takes place but not how much |
| NEW | Significant positive effects | No other transport options | More convenient to walk or cycle as infrastructure goes where it’s needed which encourages use and leads to more people seen to be active |  |  |
| **Improves connectivity/directness** |  |  |  |  |  |
| *a) Connecting destinations* |  |  |  |  |  |
| iC | Significant positive effects | Fragmented route network | More convenient to walk/cycle, people use the infrastructure and may explain increases in walking and cycling | Existing cyclists | May change where cycling takes place but not how much |
| PACE | Inconclusive or no effect | Poor street connectivity | More convenient to walk/cycle, people use the infrastructure and may explain increases in walking and cycling |  |  |
| KNOX | Significant positive effects | Poor street connectivity  Inactive population | More convenient to walk/cycle, people use the infrastructure and may explain increases in walking and cycling |  |  |
| SYD | Significant positive effects |  |  | Existing cyclists | Reduces journey times which encourages use but could decrease cycling time |
| DUR | Inconclusive or no effect | Largely urban area with a good cycle network | More comprehensive/longer routes possible and may encourage use of trail | Already feel safe  Those who are inactive | No perceived need which may explain low usage |
| PORT | Inconclusive or no effect | Existing cyclists | Longer routes become cyclable and more convenient which may lead to increases in cycling time | Existing cyclists | In the wrong direction or not where needed which may lead to no change in cycling time |
| *b) Network continuity* |  |  |  |  |  |
| DEL | Positive effect of uncertain significance | Coherent cycle network and supportive social conditions for cycling but some physical barriers  Where travel time is the biggest influence on mode of travel | Provided important missing links and greater route choice, cycling becomes a viable option and faster than before which led to increases in cycling trips  Creates logical and predictable routes with an easy mental map and can easily assess distance and travel time, which may mean cycling becomes a more viable option, greater route choice and cycling used for more trips |  |  |
| MIN | Significant positive effects | Good infrastructure provision  Existing cyclists | Provided important missing links and faster, more direct routes, increases in commuting trips by bike  More convenient/more direct routes which may encourage use of the infrastructure | Residents in city centre or those travelling long distances | Infrastructure may not go where it’s needed (or connect with essential destinations) which may led no observed changes in mode share. |
| **(ii) SAFETY** |  |  |  |  |  |
| **Segregation from motor vehicles** |  |  |  |  |  |
| iC | Significant positive effects | Busy car dominated urban environment, some with high speed and volume roads | Alleviate concerns about traffic safety which may encourage use of infrastructure for walking/ cycling | Space is used as location for crime or antisocial behaviour | May generate concerns about personal safety which could lead to low usage |
| DEL | Positive effect of uncertain significance | Busy car dominated urban environment, some with high speed and volume roads | Reduces conflict between motorists and cyclists which may explain increasing numbers of journeys made by bike and fewer accidents |  |  |
| MIN | Significant positive effects | Busy car dominated urban environment, some with high speed and volume roads | Easier and safer to cycle which means routes may be used for commuting |  |  |
| SYD | Significant positive effects |  |  | Some parts of a route remain unsafe | Not safe to walk/cycle on all parts of the journey which may explain low usage |
| DUR | Inconclusive or no effect | For existing cyclists | Safer place to cycle away from traffic which may encourage use of trail |  |  |
| GLAS | Positive effect of uncertain significance | Existing on-road cycle paths | Reduces conflict between motorists and cyclists and improves safety on route (if infrastructure provides missing link) and may lead to use of the infrastructure | Existing cyclists | May change where cycling takes place but not how much |
| PORT | Inconclusive or no effect |  |  | Those who already don’t walk or cycling might not still feel safe | Other barriers to take up and potentially no change in levels of walking or cycling |
| BIXI |  |  |  | Low levels of cycling and therefore motorists have little experience of sharing road space with cyclists | Cycling still perceived as a risky activity |
| **Reduces perception of crime** |  |  |  |  |  |
| iC | Significant positive effects | For vulnerable groups | Alleviate concerns about personal safety which may encourage use of infrastructure | If intervention does not reduce perceived risk | Few people may use the infrastructure |
| BIXI | Significant positive effects | Fear of bike theft | Makes using a bike and leaving in public safer and encourages use | For existing cyclists | Might change how and where they cycle, but potentially not increase the duration |
| **(iii) QUALITY OF EXPERIENCE** |  |  |  |  |  |
| KNOX | Significant positive effects | In dense urban neighbourhood | May provide a more pleasant place for recreational walking which may lead to more walking |  |  |
| PACE |  | Poor street conditions for walking | More pleasant to walk encourages more people to be active in the neighbourhood and changes norms for walking |  |  |
| PORT |  |  |  | For those already walking or cycling | More pleasant to walk and may change where cycling takes place but not how much |
| DUR |  |  |  | For those already walking or cycling | More pleasant to walk and may change where cycling takes place but not how much |
| DEL |  |  |  | For business trips or commute to work | Smoother ride and more comfortable to cycle which may lead to more commuting trips by bike |

Information from one study might provide information about reasons for success or otherwise s
